# Chemo-Enzymatic Synthesis of Chiral Epoxides Ethyl and Methyl (*S*)-3-(Oxiran-2-yl)propanoates from Renewable Levoglucosenone: An Access to Enantiopure (*S*)-Dairy Lactone

**DOI:** 10.3390/molecules21080988

**Published:** 2016-07-29

**Authors:** Aurélien A. M. Peru, Amandine L. Flourat, Christian Gunawan, Warwick Raverty, Martyn Jevric, Ben W. Greatrex, Florent Allais

**Affiliations:** 1 Chaire ABI—AgroParisTech, CEBB, 3 rue des Rouges Terres, 51110 Pomacle, France; florent.allais@agroparistech.fr (A.A.M.P.); florent.allais@agroparistech.fr (A.L.F.); 2 Institut Jean-Pierre Bourgin, INRA, AgroParisTech, CNRS, Université Paris-Saclay, RD10, 78026 Versailles Cedex, France; 3 Circa Group Pty Ltd., 34 Norkfolk Court, Coburg, 3058 Victoria, Australia; christian.gunawan@circagroup.com.au (C.G.); warwick.raverty@circagroup.com.au (W.R.); 4 School of Science and Technology, University of New England, Armidale, 2350 New South Wales, Australia; jevric.m@gmail.com; 5 UMR 782 GMPA, INRA, AgroParisTech, CNRS, Université Paris-Saclay, Avenue Lucien Brétignières, 78850 Thiverval-Grignon, France

**Keywords:** epoxide, flavor, levoglucosenone, chirality, total synthesis

## Abstract

Chiral epoxides—such as ethyl and methyl (*S*)-3-(oxiran-2-yl)propanoates ((***S***)**-1a**/**1b**)—are valuable precursors in many chemical syntheses. Until recently, these compounds were synthesized from glutamic acid in four steps (deamination, reduction, tosylation and epoxide formation) in low to moderate overall yield (20%–50%). Moreover, this procedure requires some harmful reagents such as sodium nitrite ((eco)toxic) and borane (carcinogen). Herein, starting from levoglucosenone (**LGO**), a biobased chiral compound obtained through the flash pyrolysis of acidified cellulose, we propose a safer and more sustainable chemo-enzymatic synthetic pathway involving lipase-mediated Baeyer-Villiger oxidation, palladium-catalyzed hydrogenation, tosylation and treatment with sodium ethoxide/methoxide as key steps. This route afforded ethyl and methyl (*S*)-3-(oxiran-2-yl)propanoates in 57% overall yield, respectively. To demonstrate the potentiality of this new synthetic pathway from **LGO**, the synthesis of high value-added (*S*)-dairy lactone was undertaken from these epoxides and provided the target in 37% overall yield from **LGO**.

## 1. Introduction

**Figure 1:**
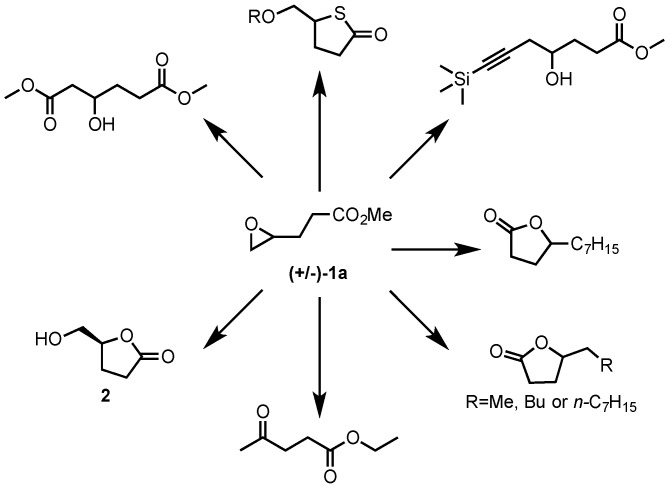
Some chemical intermediates synthesized from epoxide (+/−)-**1a**.

Chiral epoxides are widely used as intermediates in organic synthesis. For example, methyl (*S*)-3-(oxiran-2-yl)propanoate (**1a**) has been employed for the synthesis of many compounds such as elicitor [1], Streptrubin B [2] or chiral β-3-substituted homopropargyl [3], thiobutyrolactone [4], γ-hydroxyesters [5], diol-γ or δ-lactones [6], keto-esters [7], (+/−)-4-alkanolides [8] (Figure 1).

**Scheme 1:**
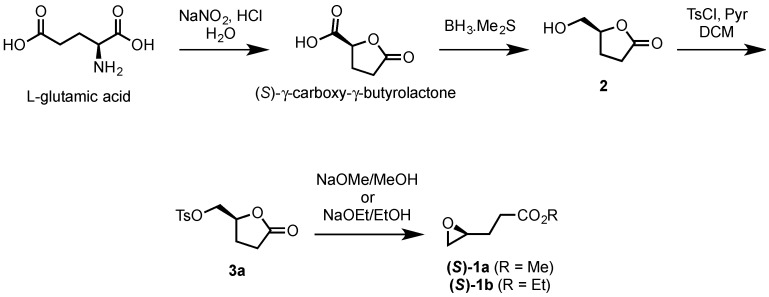
Synthesis of epoxides **(*S*)-1a/1b** from l-glutamic acid [3,17].

Classically, epoxides such as **1a** are obtained from (*S*)-γ-hydroxymethyl-γ-butyrolactone (**2H-HBO**, **2**), itself derived from glutamic acid (Scheme 1) [9,10,11,12,13,14,15,16,17,18,19], a natural amino acid that is produced by fermentation on a commercial scale [20]. Glutamic acid is first deaminated to give the intermediate (*S*)-γ-carboxy-γ-butyrolactone, which was first reported by Austin et al. using nitrous acid [11]. The protocol then evolved in 1978 with the use of a light excess of sodium nitrite and HCl instead of nitrous acid [12] and, to the best of our knowledge, remains unchanged today [13]. Three different routes have been reported for the reduction of (*S*)-γ-carboxy-γ-butyrolactone into **2**. The first one is a 2-step procedure involving the esterification of the carboxylic acid followed by its reduction with NaBH_4_ [12,14,15,16]. The second route is quite similar but uses an acid chloride instead of the carboxylic acid [10]. Finally, the third and last route consists of a one-step reduction of the carboxylic acid into the alcohol in presence of borane-methylsulfide (BH_3_-Me_2_S) [3,17] or borane-THF [18]. It is noteworthy that the substitution of borane-methylsulfide by borane-THF to obviate odor problems on large scale [12,19] resulted in over-reduction to the lactol [16]. Although these three routes provide **2** in good yields, they either require two steps or use a carcinogenic reagent (i.e., BH_3_-Me_2_S). The epoxides are then obtained via the tosylation of **2** followed by treatment with sodium alkoxide.

**Scheme 2:**
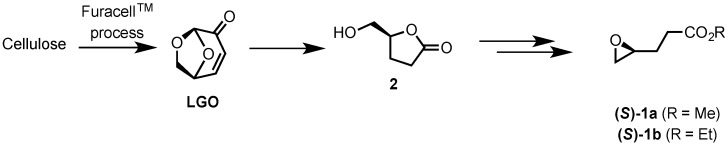
Synthesis of epoxides (***S***)**-1a/1b** from levoglucosenone (**LGO**).

Recently, we reported on the eco-friendly synthesis of **2** by a chemo-enzymatic process from levoglucosenone (**LGO**) [21,22,23,24,25] a valuable chiral chemical platform obtained from flash pyrolysis of cellulosic residues, such as softwood sawdust (Furacell™ process) [26]. Providing **2** in very good yield and high purity through a lipase-based mediated oxidation, this sustainable synthetic pathway has been studied further by our group as an alternative to the one involving l-glutamic acid to achieve the synthesis of epoxides (***S***)**-1a** and (***S***)**-1b** (Scheme 2).

**Scheme 3:**
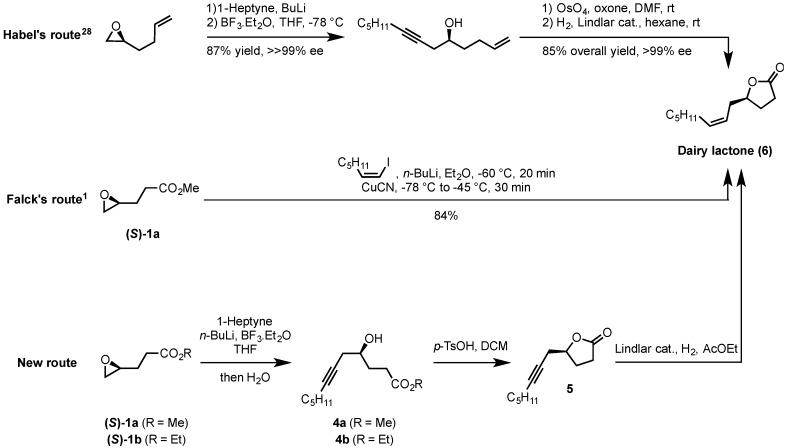
Synthetic routes to (*S*)-dairy lactone (**6**).

Dairy lactone is the common name given by flavor specialists to (*Z*)-5-(oct-2-en-1-yl)dihydrofuran-2(3*H*)-one (**6**) (CAS 18679-18-0) of unspecified enantiomeric purity. This lactone, which occurs naturally in cows’ milk and also the mycelia of *Fusarium poae,* exhibits a very fruity nuanced odor (also described as fatty, waxy) as well as a creamy and dairy-like taste, hence its name [27,28]. Dairy lactone is commonly used in as flavor for bakery and milk products at up to 1 ppm, imitation dairy at 5 ppm, and fruit sorbet at 1 ppm [29]. Despite its very attractive flavoring properties, commercial production of dairy lactone remains relatively low (ca. 50 kg/year worldwide). Indeed, not only is it produced using a costly fermentation process (dairy lactone is sold at ca. 10,000 €/kg) [30] but some customers also complain of a rancid aftertaste in some batches of the flavor. Under these considerations, an enantioselective synthetic route to individual *S* and *R* enantiomers of **6** that leads to the commercial production of pure enantiomers is attractive as a solution to these problems (i.e., production cost and aftertaste). While the enantiomeric composition of commercial dairy lactone appears not to have been published, there is at least one publication that states that the *S*-enantiomer from *Fusarium poae* is some 3 times stronger in stimulating olefactory response in humans than the *R*-enantiomer [28]. Two synthetic pathways have been reported for the preparation of (*S*)-dairy lactone (Scheme 3). The first one, reported by Habel et al. [31] starts from an enantiopure glycidyl alkene and involves an alkynylation with heptynyl lithium followed by an osmium tetroxide-mediated oxidative cleavage of the double bound to form the lactone, and finally a *syn*-hydrogenation of the triple bound. The second pathway developed by Falck consists of the addition of the high order cuprate generated from (*Z*)-1-iodo-hept-1-ene onto the epoxides **1a** [1,32,33]. Considering that (*Z*)-1-iodo-hept-1-ene needs to be synthesized prior to cuprate formation and that its synthesis involves the use of CuCN and low temperature (−78 °C), the nucleophilic addition of lithiated 1-heptyne on glycidyls (***S***)**-1a**/**1b** followed by a *syn*-hydrogenation of the triple bound (as described in scheme 3—New route), which is a simpler and safer way to perform this synthesis, has been devised and is described herein.

The efficiency (in terms of yield) and green aspects of these new synthetic pathways to ethyl and methyl (*S*)-3-(oxiran-2-yl)propanoates as well as (*S*)-dairy lactone will be discussed with regard to the yields and green aspects of the route involving l-glutamic acid.

## 2. Results and Discussion

Herein, we report the use of **LGO** as starting material to produce ethyl and methyl (*S*)-3-(oxiran-2-yl)propanoates (**1a** and **1b**). (*S*)-Dairy lactone **6**, a potentially high-value commercial flavor chemical, was then synthesized from the two epoxides via an efficient and straightforward pathway.

### 2.1. Synthesis of Epoxides from Levoglucosenone (***LGO***)

#### 2.1.1. Synthesis of (*S*)-γ-Hydroxymethyl-γ-butyrolactone (**2**)

**Scheme 4:**
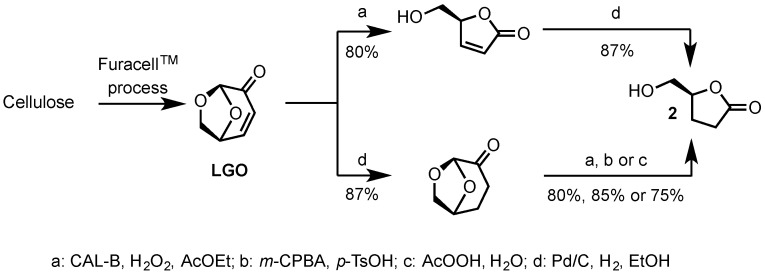
Syntheses of (*S*)-γ-hydroxymethyl-γ-butyrolactone (**2**) from cellulose.

The synthesis of the epoxides (***S***)**-1a** and (***S***)**-1b** started with the preparation of key intermediate (*S*)-γ-hydroxymethyl-γ-butyrolactone (**2**) from **LGO** using the two chemo-enzymatic pathways we previously developed and optimized [24,25] (Scheme 4). The first one consisted in performing a palladium-catalyzed hydrogenation of **LGO** (87%) followed by the Baeyer-Villiger oxidation of the resulting saturated **LGO** (**2H-LGO,** or Cyrene™) in ethyl acetate and in the presence of hydrogen peroxide and CAL-B (aka Novozyme 435^®^, N435 or immobilized lipase *Candida antarctica* type B); the subsequent acid hydrolysis of the reaction mixture with Amberlyst 15 IR dry in ethanol then provides **2** in 65% overall yield. The second pathway involves the exact same reactions but in a reversed fashion (i.e., Baeyer-Villiger oxidation of **LGO**, acid hydrolysis and palladium-catalyzed hydrogenation) providing **2** in 70% overall yield from **LGO**.

It is noteworthy that the above Baeyer-Villiger oxidation of **LGO** can be performed either chemically—using AcOOH (75%) [34,35], zeolites (96% HPLC yield) [36] or *m*-CPBA (85%) [34,35]—or enzymatically with a lipase (80%) [24,25]. Even though the *m*-CPBA- and zeolites-based Baeyer-Villiger oxidations lead to slightly better yields, lipase-mediated Baeyer-Villiger oxidation is preferred because it avoids not only the use of potentially explosive organic peroxide and harmful organic solvent (dichloromethane), but also the production of stoichiometric amount of by-product (i.e., *m*-chlorobenzoic acid). In addition, the enzyme is used in catalytic amount and can be easily recycled by simple filtration [24,25].

#### 2.1.2. Synthesis of Ethyl and Methyl (*S*)-3-(Oxiran-2-yl)propanoates ((***S***)**-1a** and ((***S***)**-1b**)

**Scheme 5:**
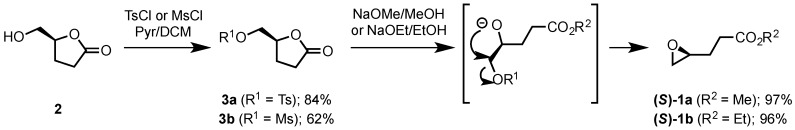
Syntheses of epoxides (***S***)**-1a/1b** from (*S*)-γ-hydroxymethyl-γ-butyrolactone (**2**).

Ethyl and methyl (*S*)-3-(oxiran-2-yl)propanoates ((***S***)**-1a** and ((***S***)**-1b**) were synthesized in two steps from **2** (Scheme 5). The primary hydroxyl moiety in compound **2** was first activated through tosylation (**3a**, 84%) and mesylation (**3b**, 64%) in pyridine/dichloromethane with TsCl and MesCl, respectively. Epoxides (***S***)**-1a** and (***S***)**-1b** were then efficiently obtained by treating the activated lactones with sodium methoxide or ethoxide. The mechanism involves two steps: (1) nucleophilic attack of sodium methoxide or ethoxide on the lactone to form the corresponding secondary alcoholate intermediate; and (2) S_N2_ intramolecular substitution of the tosylate/mesylate by the alkoxide oxygen. Crude methyl-4,5-epoxypentanoate ((***S***)**-1a**) and ethyl-4,5-epoxypentanoate ((***S***)**-1b**) were thus obtained in 97% and 96% yields from **3a** and in 42% and 86% yields from **3b**, respectively. Once purified by distillation, the optical activity of (***S***)**-1a** and (***S***)**-1b** were measured and proved in accordance with those already reported in the literature (see Materials and Methods section), demonstrating that no epimerization occurs in the process. In terms of atom economy, using mesylate **3b** would be the greener option; however, tosylate **3a** appears as a better choice as it was obtained in better yield, is crystalline allowing for simple purification and also proved more reactive in the next step. Alternatively, if sodium methoxide and ethoxide give the same yield, the latter will be preferred due to the lower toxicity of ethanol compared to methanol.

Starting from **LGO**, the most efficient synthetic route in terms of yield is the one involving tosyl chloride and sodium methoxide (81% overall yield). The greener route, that involves mesyl chloride and sodium ethoxide provides the chiral epoxides in 60% overall yield.

### 2.2. Synthesis of (S)-Dairy Lactone

**Scheme 6:**
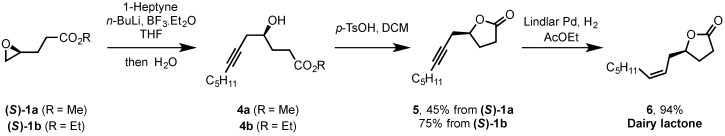
Synthesis of (*S*)-dairy lactone from epoxide (***S***)**-1a/1b**.

(*S*)-Dairy lactone was thus synthesized in three steps from epoxides: (1) regioselective opening of the epoxides, (2) acid-mediated lactonization, and (3) *syn*-stereoselective hydrogenation (Scheme 6). The Lewis acid-mediated opening of the epoxide in the presence of lithiated hept-1-yne resulted in the addition of the latter on the less substituted carbon, providing γ-hydroxyesters **4a** and **4b**. Acid-catalyzed lactonization of crude **4a** or **4b** was then performed in presence of *p*-toluenesulfonic acid to give lactone **5** in 45% and 74% yields, respectively. Finally, (*S*)-dairy lactone **6** was obtained via *syn*-selective hydrogenation of the alkyne moiety by using Lindlar palladium catalyst (94% yield). In summary, (*S*)-dairy lactone was successfully synthesized in seven steps and in 37% overall yield from **LGO** (69% overall yield from (***S***)**-1a**/(***S***)**-1b**).

## 3. Materials and Methods

Levoglucosenone (**LGO**) was kindly supplied by Circa Group. Novozyme 435^®^ (aka N435, immobilized lipase *Candida antarctica* type B or CAL-B) was purchased from Univar. Other reagents were purchased from Sigma-Aldrich and used as received. Solvents were purchased from ThermoFisher Scientific. When necessary, they were dried on mBraun SPS 800. CDCl_3_ was purchased from Euriso-top. Evaporations were conducted under reduced pressure at temperature below 40 °C. Column chromatography was carried out with an automated flash chromatography (PuriFlash 4100, Interchim) and pre-packed INTERCHIM PF-30SI-HP (30 µm silica gel) columns. IR and UV analyses were performed on Cary 630 FTIR and Cary 60 UV-Vis from Agilent technologies, respectively. NMR analyses were recorded on a Bruker Fourier 300. ^1^H-NMR spectra of samples were recorded in CDCl_3_ at 300 MHz, chemical shifts were reported in parts per million relative to residual solvent peak (δ = 7.26 ppm). ^13^C-NMR spectra of samples were recorded at 75 MHz (CDCl_3_ residual signal at δ = 77.16 ppm).

### 3.1. Chemo-Enzymatic Synthesis of (S)-γ-Hydroxymethyl-γ-butyrolactone (***2***)

N435 (237 PLU/mmol of **LGO**) was suspended in ethyl acetate (210 mL, *c* = 0.75 M), then **LGO** (20 g, 159 mmol) and finally H_2_O_2_ (30% *w*/*w* in water, 18 mL, 159 mmol, 1 equiv) were added. The reaction mixture was incubated at 40 °C and 150 rpm during 16 h. Then, N435 was filtered off and ethyl acetate was removed under reduced pressure. Crude residue was diluted with ethanol (40 mL) and added to a suspension of Amberlyst 15 IR dry (40 g) in ethanol (80 mL). After a night of incubation at 37 °C and 150 rpm, Amberlyst was filtered off. The crude mixture was flushed with nitrogen and 10% *w* palladium on carbon (2 g) was added, the reaction was then stirred at atmospheric pressure under hydrogen flux for a night. The crude mixture was filtered through a pad of Celite^®^ and the filtrate was concentrated to dryness. The resulting orange oil was purified by silica gel chromatography (elution with 50 to 100% ethyl acetate in cyclohexane) to yield pure **2H-HBO** (**2**) as a colorless oil (70%); [α${]}_{\text{D}}^{20}$ +52.9° (*c* 0.01, CHCl_3_); Lit. [26] +55.2° (*c* 0.1, CHCl_3_); UV (EtOH) λ_max_ 207 nm; FT-IR (neat, cm^−^^1^): 3420 (OH), 2938, 1752 (C=O), 1353, 1181; ^1^H-NMR (CDCl_3_, 300 MHz): δ 2.20 (m, 2H, H-3), 2.61 (m, 3H, H-2a, H-2b, OH), 3.66 (dd, 1H, *J* = 12.6 and 4.5 Hz, H-5a), 3.92 (dd, 1H, *J* = 12.6 and 2.7 Hz, H-5b), 4.64 (m, 1H, H-4); ^13^C-NMR (CDCl_3_, 75 MHz): δ 23.1 (t, C-2), 28.7 (t, C-3), 64.1 (t, C-5), 80.8 (d, C-4), 177.7 (s, C-1); HRMS: *m/z* [M + Na]^+^ calcd for C_5_H_8_NaO_3_: 139.0371, found: 139.0379.

### 3.2. Synthesis of ***2*** Using m-CPBA

To a stirred solution of dihydrolevoglucosenone (4.0 g, 31.2 mmol) in dichloromethane (50 mL) cooled using a water bath was added 70% *meta-*chloroperbenzoic acid (11.5 g, 47 mmol). *p*-TsOH (800 mg, 5 mmol) was then added and the resulting solution stirred for 16 h. Volatiles were removed under reduced pressure and then 1 M HCl (20 mL) was added and the resulting solution stirred overnight. The mixture was filtered, the precipitate washed with water and the filtrate concentrated under reduced pressure. Purification of the residue by flash chromatography to remove residual *m-*chloroperbenzoic acid afforded **2** as colorless crystals (3.07 g, 85%).

### 3.3. Synthesis of (S)-γ-Tosyloxymethyl-γ-butyrolactone (***3a***)

Under nitrogen, **2** (2.5 g, 21.5 mmol) and *p*-toluenesulfonyl chloride (6.16 g, 32.3 mmol, 1.5 equiv) were dissolved in a mixture of dry dichloromethane (10 mL) and pyridine (4.3 mL) and stirred at room temperature for 9 h. The reaction mixture was diluted with dichloromethane (100 mL), washed thrice with HCl solution (3 M, 50 mL), and twice with a saturated solution of NaHCO_3_ (50 mL) and with brine (50 mL). The organic layer was then dried over anhydrous MgSO_4_, filtered and concentrated. The crude mixture was triturated in Et_2_O (20 mL) to yield **3a** as a beige powder (4.88 g, 84%); m.p.: 83.3 °C; [α${]}_{\text{D}}^{20}$ +47.9° (*c* 0.11, CHCl_3_); Lit. [7] +44.5° (*c* 0.95, CHCl_3_); UV (CDCl_3_) λ_max_ 237, 263, 274 nm; FT-IR (neat,): 2961, 1765 (C=O), 1595 (C=Carom), 1448 (C=Carom), 1364, 1169 cm^−^^1^; ^1^H-NMR (CDCl_3_, 300 MHz): δ 2.25 (m, 2H, H-3), 2.45, (s, 3H, H-10), 2.55 (m, 2H, H-2), 4.16 (m, 2H, H-5), 4.68 (m, 1H, H-4), 7.36 (d, 2H, *J* = 8.1 Hz, H-8), 7.78 (d, 2H, *J* = 8.1 Hz, H-7); ^13^C-NMR (CDCl_3_, 75 MHz): δ 21.8 (q, C-10), 23.7 (t, C-2), 28.0 (t, C-3), 70.1 (t, C-5), 76.5 (d, C-4), 128.1 (d, C-7), 130.2 (d, C-8), 132.3 (s, C-9), 145.6 (s, C-6), 176.2 (s, C-1).

### 3.4. Synthesis of (S)-γ-Mesyloxymethyl-γ-butyrolactone (***3b***)

Under nitrogen, **2** (5.0 g, 43.1 mmol) and methanesulfonyl chloride (5.0 mL, 64.7 mmol, 1.5 equiv), were dissolved in a mixture of dry dichloromethane (22 mL) and pyridine (8.7 mL) and stirred at room temperature for 3 h. The reaction mixture was diluted with dichloromethane (200 mL), washed thrice with HCl solution (3 M, 100 mL), twice with a saturated solution of NaHCO_3_ (100 mL) and with brine (100 mL). The organic layer was then dried over anhydrous MgSO_4_, filtered and concentrated. The crude mixture was triturated in Et_2_O (40 mL) to yield **3b** as a beige powder (5.2 g, 62%); m.p.: 46.5 °C; [α${]}_{\text{D}}^{20}$ +30.6° (*c* 0.10, CHCl_3_); Lit. [15] +33.7° (*c* 1, CHCl_3_); FT-IR (neat): 3012, 2936, 1758 (C=O), 1338, 1157 cm^−1^; ^1^H-NMR (CDCl_3_, 300 MHz): δ 2.12 (m, 1H, H-3a), 2.36 (m, 1H, H-3b), 2.56 (m, 2H, H-2), 3.05 (s, 3H, H-6), 4.26 (m, 1H, H-5a), 4.42, (m, 1H, H-5b), 4.75 (m, 1H, H-4); ^13^C-NMR (CDCl_3_, 75 MHz): δ 23.4 (t, C-3), 28.0 (t, C-2), 37.7 (q, C-6), 70.0 (t, C-5), 76.8 (d, C-4), 176.3 (s, C-1).

### 3.5. Synthesis of (S)-Methyl 4,5-epoxypentanoate (*(**S**)**-1a***) and (S)-Ethyl 4,5-epoxypentanoate (*(**S**)**-1b***)

Under nitrogen, **3a** (2.0 g, 7.37 mmol) or **3b** (1.4 g, 7.37 mmol) was dissolved in MeOH/EtOH (10 mL) and sodium methoxide (400 mg, 1.1 equiv)/sodium ethoxide (510 mg, 1.1 equiv) was added. The reaction was stirred at room temperature for 3 h and then the solvent was evaporated and water (10 mL) was added. The aqueous layer was extracted twice with Et_2_O (15 mL), the organic layers were combined, washed with brine (20 mL), dried over anhydrous MgSO_4_ and concentrated to dryness.

(***S***)**-1a** (0.93 g, 97%) orange oil. A small quantity of the product was distilled under reduced pressure to give a colorless oil. [α${]}_{\text{D}}^{20}$ −17.0° (*c* 0.11, CHCl_3_); Lit. [7] −17.9° (*c* 7.4, CHCl_3_); FT-IR (neat): 2952, 1731 (C=O), 1437, 1360, 1256, 1172 cm^−1^; ^1^H-NMR (CDCl_3_, 300 MHz): δ 1.78 (m, 1H, H-3a), 1.98 (m, 1H, H-3b), 2.48 (m, 3H, H-2, H-5a), 2.77 (m, 1H, H-5b), 2.98 (m, 1H, H-4), 3.69 (s, 3H, H-6); ^13^C-NMR (CDCl_3_, 75 MHz): δ 27.7 (t, C-3), 30.3 (t, C-2), 47.2 (t, C-5), 51.4 (d, C-4), 51.8 (q, C-6), 173.4 (s, C-1).

(***S***)**-1b** (1.02 g, 96%) orange oil. A small quantity of the product was distilled under reduced pressure to give a colorless oil. FT-IR (neat, cm^−^^1^): 2982, 1729 (C=O), 1371, 1254, 1176; [α${]}_{\text{D}}^{20}$ −15.6° (*c* 0.11, CHCl_3_); Lit. [15] −17.1° (*c* 0.8, CHCl_3_); ^1^H-NMR (CDCl_3_, 300 MHz): δ 1.22 (td, 3H, *J* = 7.2 and 1.2 Hz, H-7), 1.74 (m, 1H, H-3), 1.92 (m, 1H, H-3), 2.42 (m, 3H, H-2, H-5), 2.72 (m, 1H, H-5), 2.94 (m, 1H, H-4), 4.10 (qd, 2H, *J* = 7.2 and 1.2 Hz, H-6); ^13^C-NMR (CDCl_3_, 75 MHz): δ 14.4 (q, C-7), 27.8 (t, C-3), 30.6 (t, C-2), 47.2 (t, C-5), 51.4 (d, C-4), 60.7 (t, C-6), 173.0 (s, C-1).

### 3.6. Synthesis of (S)-5-(Oct-2-yn-1-yl)-γ-butyrolactone (***5***)

Under nitrogen at −78 °C, 1-heptyne (1.0 mL, 7.53 mmol, 1.4 equiv) was dissolved in dry THF (11 mL), then *n*-butyl lithium 2.5 M in hexane (3.0 mL, 7.53 mmol, 1.4 equiv) was added. After 5 min, boron trifluoride etherate (0.93 mL, 7.53 mmol, 1.4 equiv) was added, and 30 min later, (***S***)**-1a** (0.70 g, 5.38 mmol, 1 equiv), or (***S***)**-1b** (0.78 g, 5.38 mmol, 1 equiv), was added. The reaction media was stirred at −78 °C for 3 h, then quenched with a saturated solution of NaHCO_3_ (6 mL) and water (50 mL) was added. The aqueous layer was extracted with dichloromethane (3 × 20 mL). The organic layers were combined, washed with brine, dried over anhydrous MgSO_4_ and concentrated to dryness. The crude product was diluted in dichloromethane (50 mL) and *p*-TsOH monohydrate (270 mg, 1.4 mmol, 0.25 equiv) was added. After 3 h, the reaction was quenched with a saturated solution of NaHCO_3_. The organic layer was dried over anhydrous MgSO_4_, filtered and concentrated. The orange oil was purified by silica gel chromatography (elution with 95% to 90% ethyl acetate in cyclohexane) to yield pure **5** as a yellow oil (70% from (***S***)**-1b** and 45% from (***S***)**-1a**); [α${]}_{\text{D}}^{20}$ −29.5° (*c* 0.12, CHCl_3_); FT-IR (neat, cm^−^^1^): 2929, 2858, 1772 (C=O), 1349, 1172; ^1^H-NMR (CDCl_3_, 300 MHz): δ 0.89 (m, 3H, H-12), 1.31 (m, 4H, H-10, H-11), 1.47 (m, 2H, H-9), 2.13 (m, 3H, H-3, H-8), 2.38 (m, 1H, H-3), 2.58 (m, 4H, H-2, H-5), 4.60 (m, 1H, H-4); ^13^C-NMR (CDCl_3_, 75 MHz): δ 14.1 (q, C-12), 18.7 (t, C-8), 22.3 (t, C-11), 25.6 (t, C-5), 26.6 (t, C-3), 28.6 (t, C-2, C-9), 31.1 (t, C-10), 73.6 (s, C-6), 78.2 (d, C-4), 83.7 (s, C-7), 177.0 (s, C-1).

### 3.7. Synthesis of (S)-Dairy Lactone (***6***)

**5** (2.72 g, 14.0 mmol) was dissolved in ethyl acetate (56 mL, *c* = 0.25 M) and Lindlar palladium (0.20 g, 7.5% *w*/*w*) was added. The reaction was vigorously stirred under 1 atmosphere of hydrogen until complete by GC. The crude mixture was filtered through a pad of Celite^®^ and the filtrate was concentrated to dryness. The resulting orange oil was purified by silica gel chromatography (elution with 20%–50% ethyl acetate in cyclohexane) to yield pure *S*-dairy lactone **6** (2.59 g, 94%); [α${]}_{\text{D}}^{20}$ +7.5° (*c* 0.51, CHCl_3_); Lit. [29] +17.7° (*c* 0.37, MeOH); FT-IR (neat): 2923, 2854, 1771 (C=O), 1459, 1348, 1173 cm^−1^; ^1^H-NMR (CDCl_3_, 300 MHz): δ 0.82 (m, 3H, H-12), 1.23 (m, 6H, H-9, H-10, H-11), 1.84 (m, 1H, H-3a), 1.98 (m, 2H, H-8), 2.23 (m, 1H, H-3b), 2.37 (m, 1H, H-5a), 2.47 (m, 3H, H-2, H-5b), 4.47 (q, 1H, *J* = 6.6 Hz, H-4), 5.29 (m, 1H, H-6), 5.51 (m, 1H, H-7); ^13^C-NMR (CDCl_3_, 75 MHz): δ 14.0 (q, C-12), 22.5 (t, C-10), 27.1 (t, C-3), 27.4 (t, C-8), 28.7 (t, C-2), 29.1 (t, C-11), 31.4 (t, C-9), 32.8 (t, C-5), 80.2 (d, C-4), 122.2 (d, C-6), 134.0 (d, C-7), 177.1 (s, C-1).

^1^H- and ^13^C-NMR spectra for compounds **2**, **3a**, **3b**, crude (***S***)**-1a**, crude (***S***)**-1b**, **5** and **6** can be found in Supplementary Materials.

## 4. Conclusions

A greener and more efficient synthetic method to access chiral epoxides ethyl and methyl (*S*)-3-(oxiran-2-yl)propanoates has been developed from renewable levoglucosenone using a lipase-mediated enzymatic Baeyer-Villiger oxidation. The total synthesis of (*S*)-dairy lactone has then been successfully achieved in three steps and in 69% overall yield from these chiral epoxides using alkynylation with heptynyl lithium, and Lindlar catalyst-mediated *syn*-hydrogenation as the key steps.

## Supplementary Materials

Supplementary materials can be accessed at: http://www.mdpi.com/1420-3049/21/8/988/s1.
