# Supplementary material for: Chemo-Enzymatic Synthesis of Chiral Epoxides Ethyl and Methyl (S)-3-(Oxiran-2-yl)propanoates from Renewable Levoglucosenone: An Access to Enantiopure (S)-Dairy Lactone
Source: Molecules. 2016 Jul 29;21(8):988. doi: 10.3390/molecules21080988 (PMC6272917; doi:10.3390/molecules21080988)
Supplement: Supplementary file 1 [file molecules-21-00988-s001.pdf]

# Supplementary Materials: Chemo-enzymatic Synthesis of Chiral Epoxides Ethyl and Methyl (*S*)-3-(Oxiran-2-yl)propanoates from Renewable Levoglucosenone: An Access to Enantiopure (*S*)-Dairy Lactone

**Aurélien A. M. Peru, Amandine L. Flourat, Christian Gunawan, Warwick Raverty, Martyn Jevric, Ben W. Greatrex and Florent Allais**

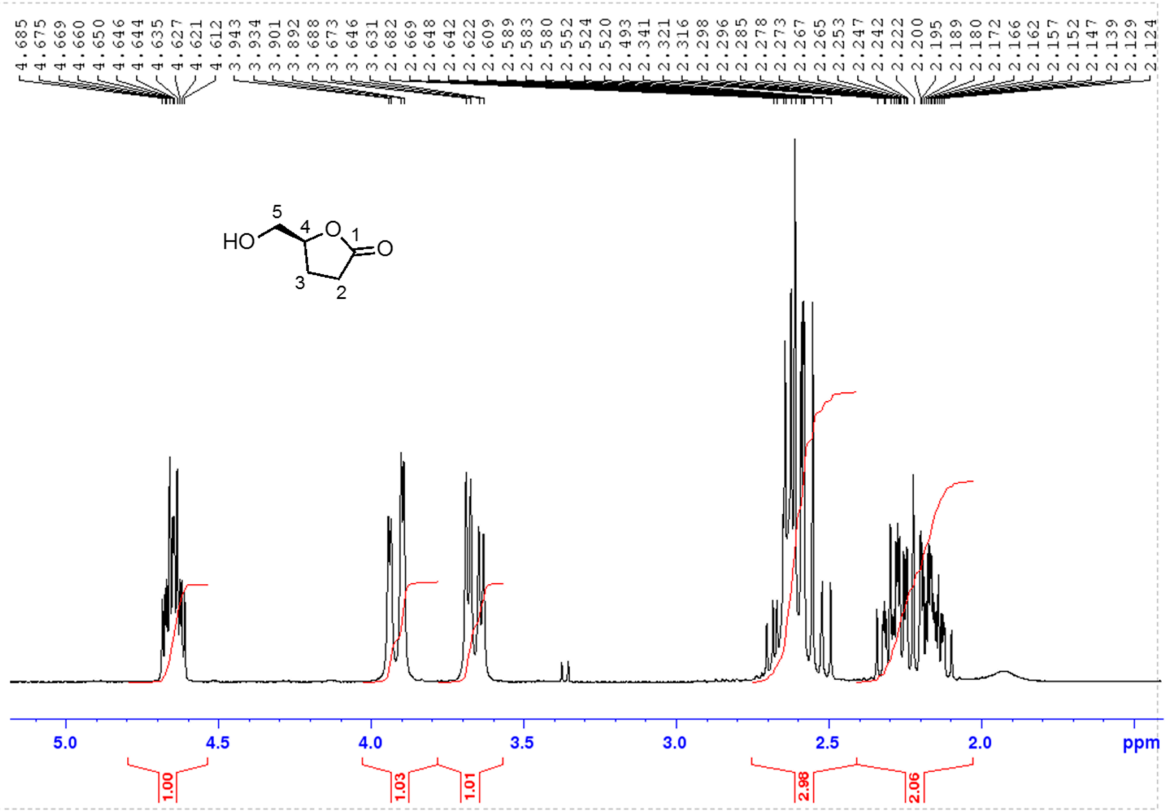


**Figure S1.** $^{1}$H-NMR spectra for (*S*)-γ-hydroxymethyl-γ-butyrolactone (**2**). $^{1}$H 300 MHz in $CDCl_3$.

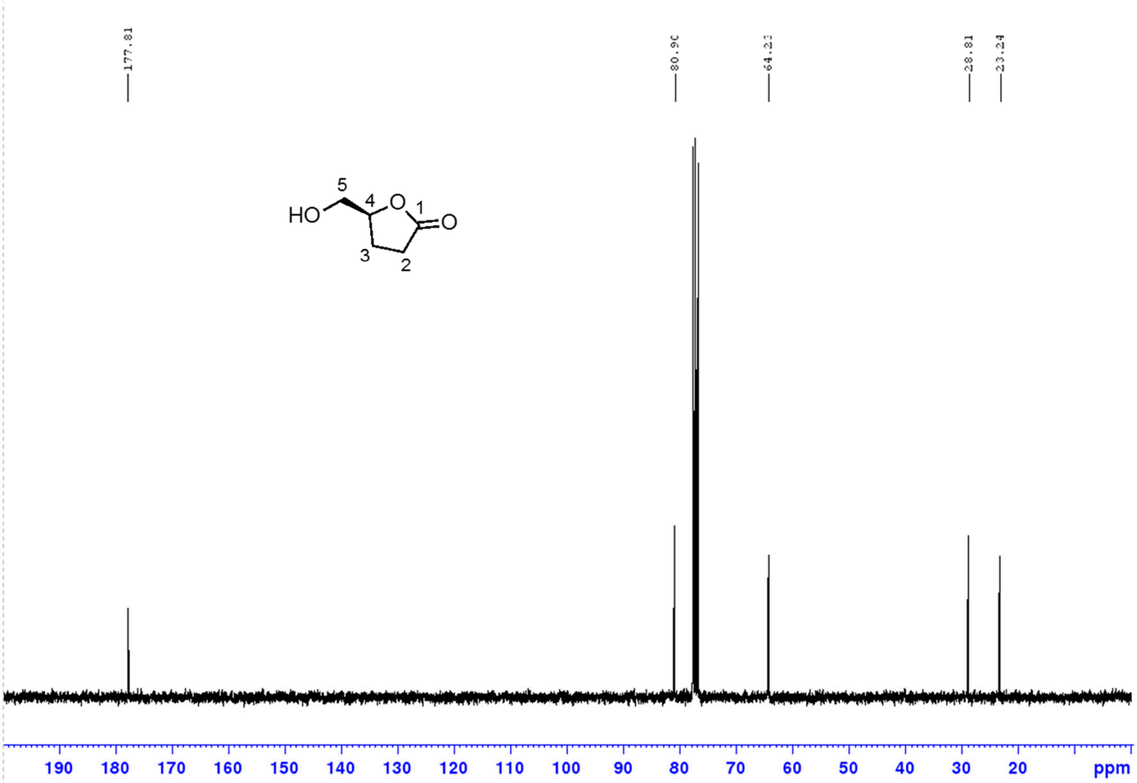


**Figure S2.** $^{13}$C-NMR spectra for (*S*)-γ-hydroxymethyl-γ-butyrolactone (**2**).$^{13}$C 75 MHz in $CDCl_3$.

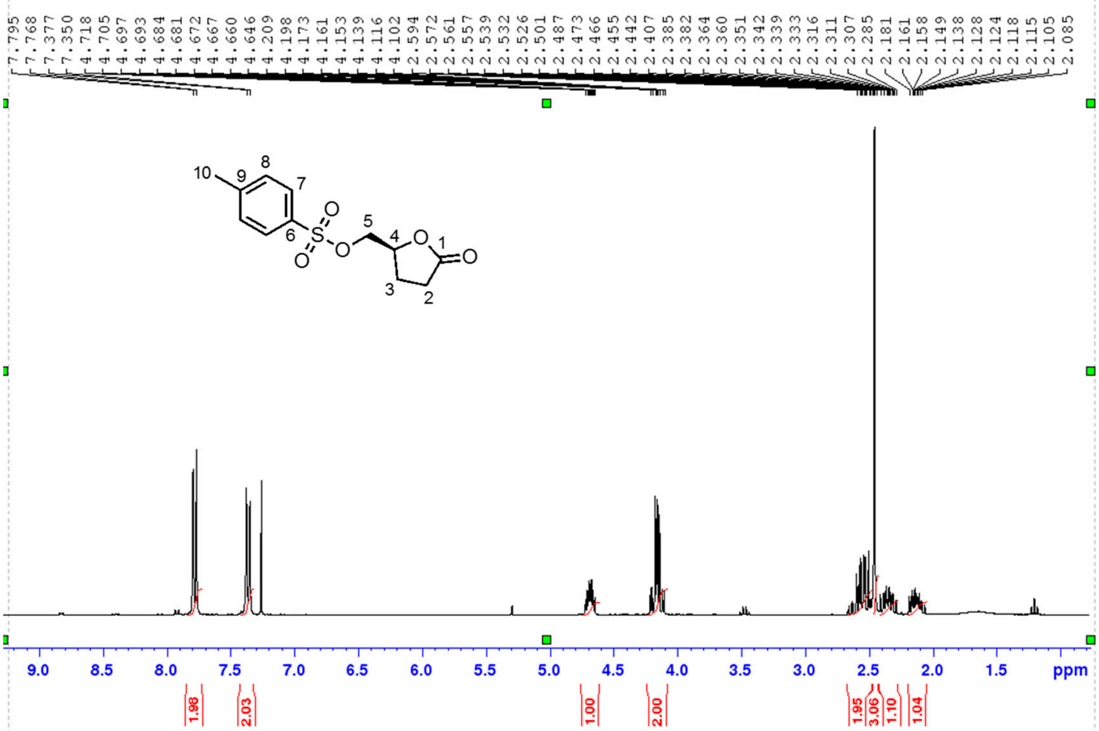


**Figure S3.** $^{1}$H-NMR spectra for (*S*)-γ-tosyloxymethyl-γ-butyrolactone (**3a**). $^{1}$H 300 MHz in $CDCl_3$.

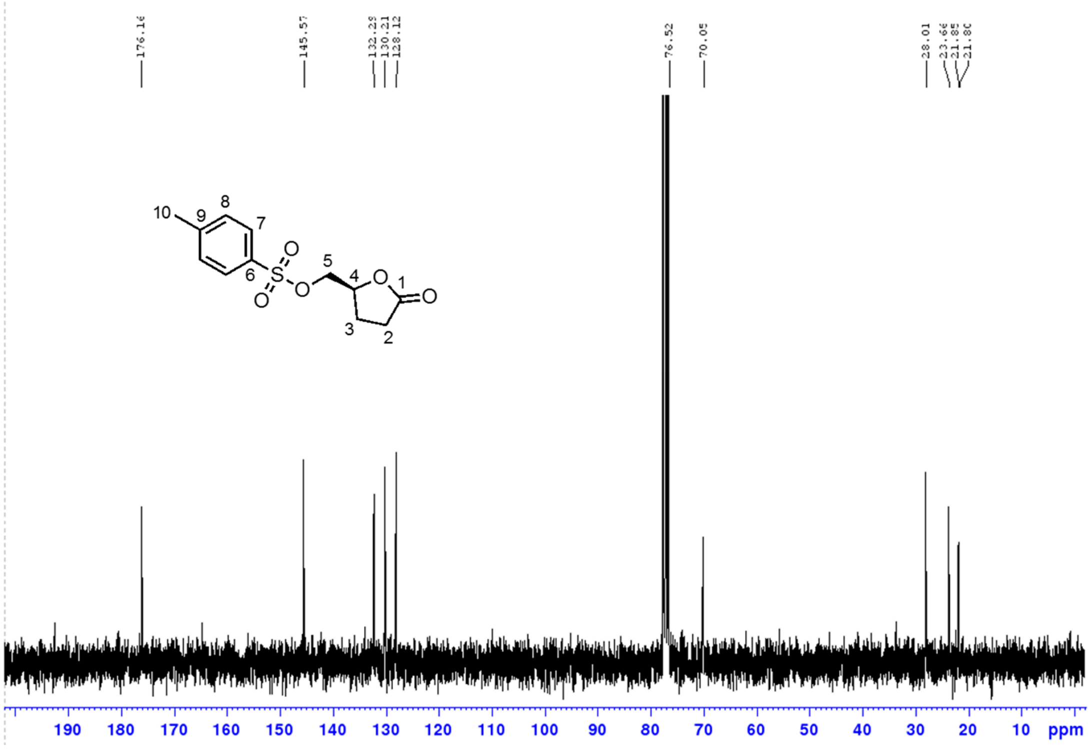


**Figure S4.** ${}^{13}$C-NMR spectra for (*S*)-γ-tosyloxymethyl-γ-butyrolactone (**3a**). ${}^{13}$C 75 MHz in $CDCl_3$.

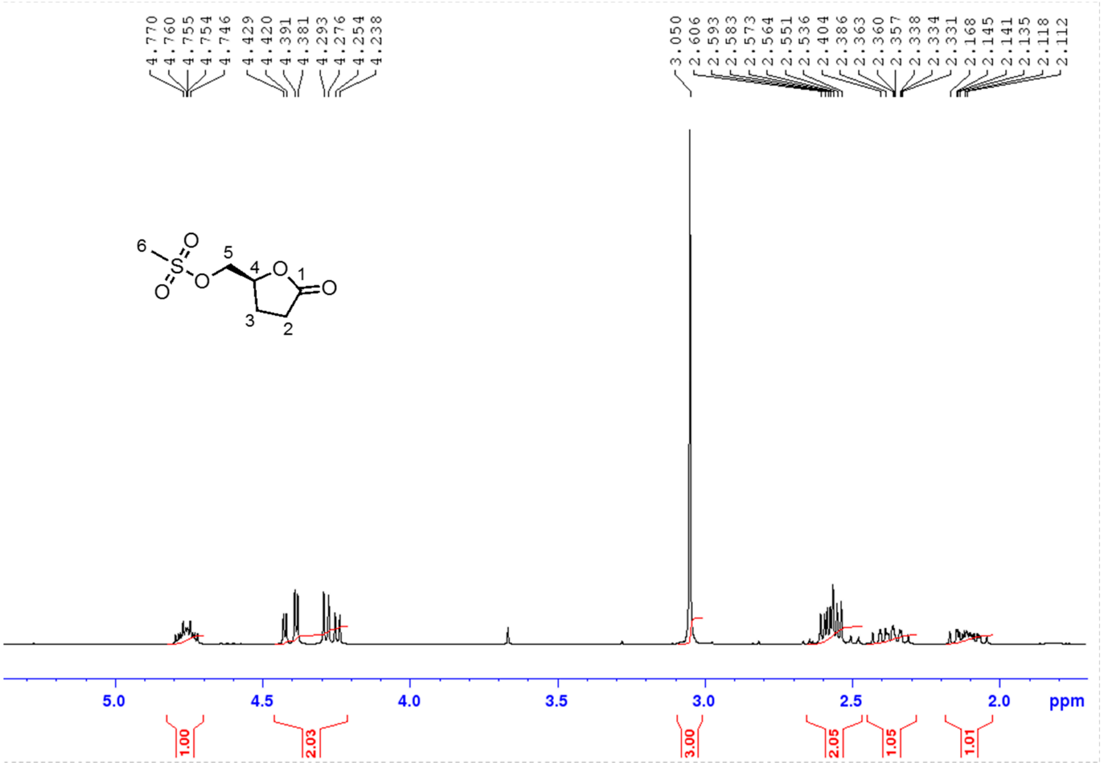


**Figure S5.** ${}^{1}$H-NMR spectra for (*S*)-γ-mesyloxymethyl-γ-butyrolactone (**3b**).${}^{1}$H 300 MHz in $CDCl_3$.

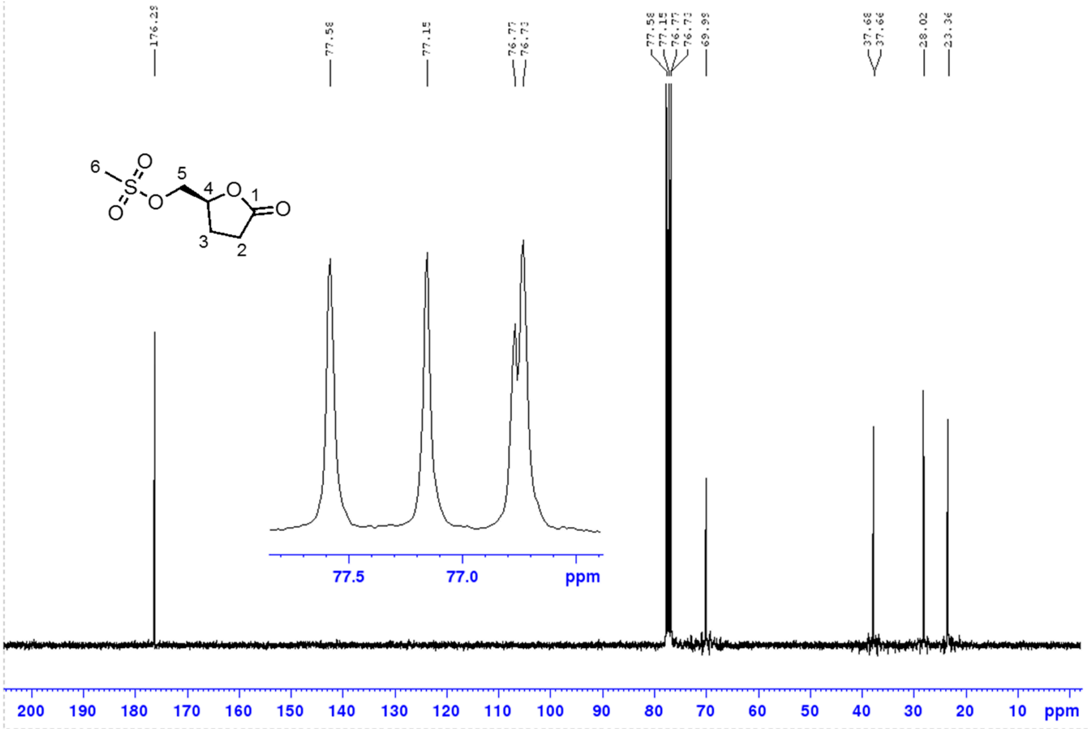

**Figure S6.** $^{13}$C-NMR spectra for (*S*)-γ-mesyloxymethyl-γ-butyrolactone (**3b**).$^{13}$C 75 MHz in $CDCl_3$.

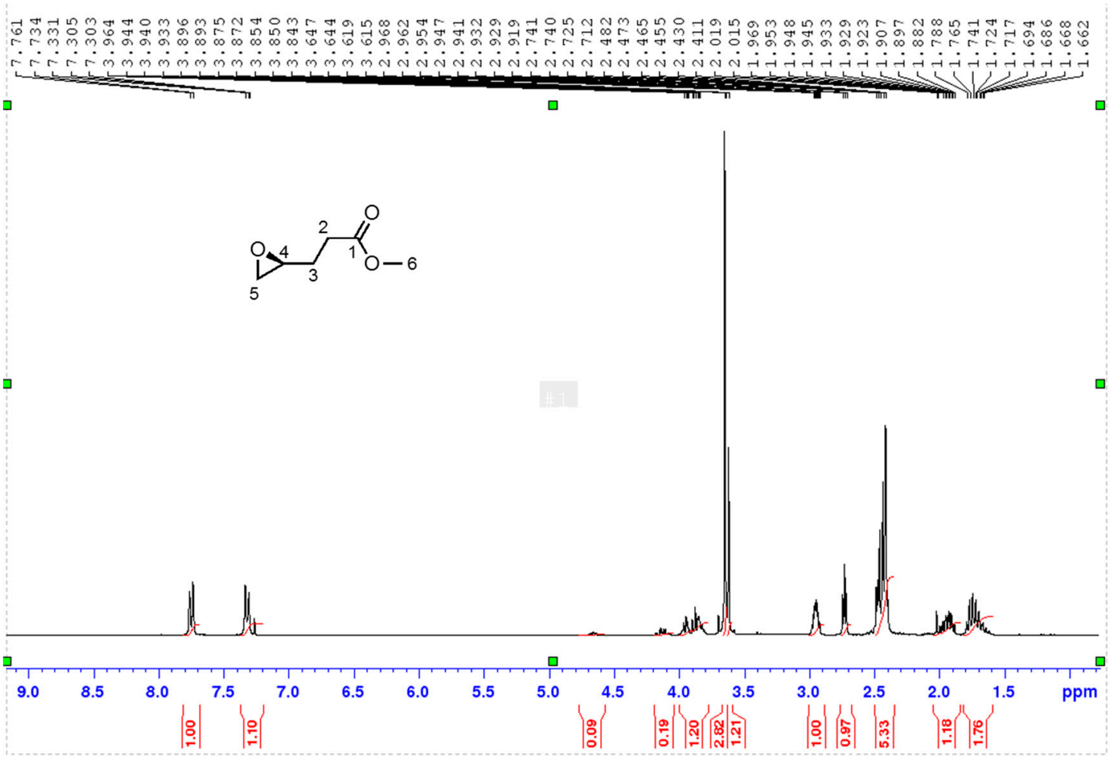

**Figure S7.** $^{1}$H-NMR spectra for (*S*)-Methyl 4,5-epoxypentanoate (**(*S*)-1a**) crude. $^{1}$H 300 MHz in $CDCl_3$.

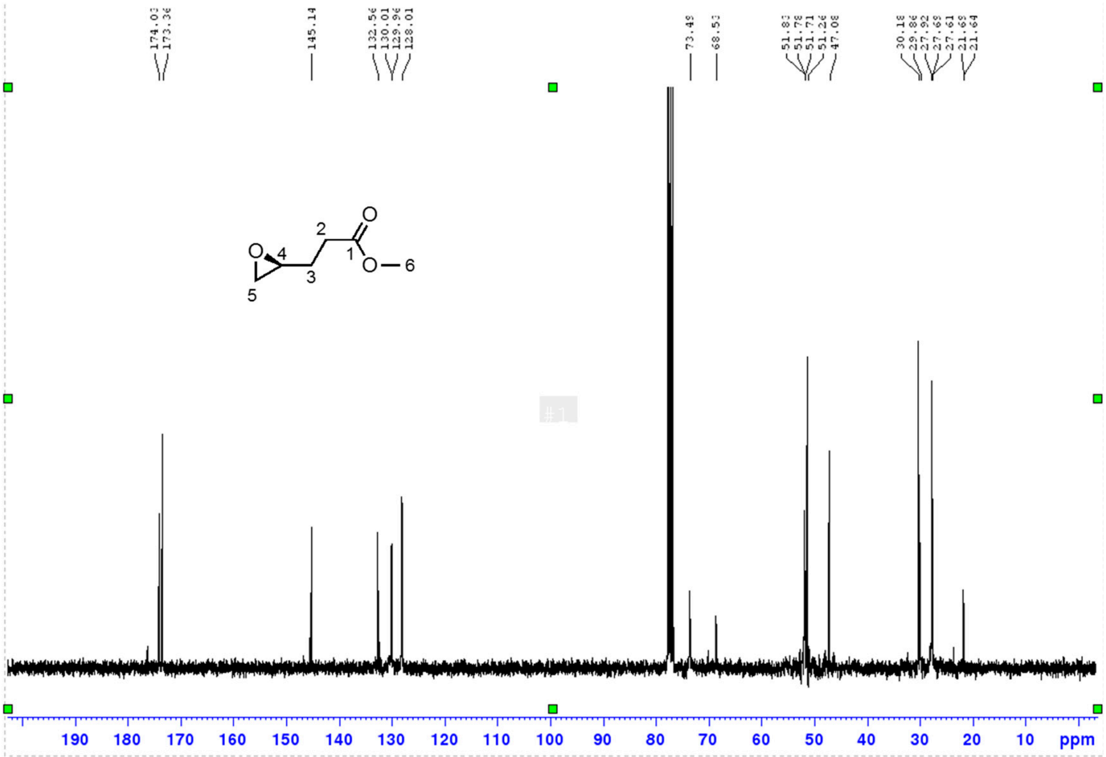

**Figure S8.** $^{13}$C-NMR spectra for (*S*)-Methyl 4,5-epoxypentanoate (**(*S*)-1a**) crude. $^{13}$C 75 MHz in $CDCl_3$.

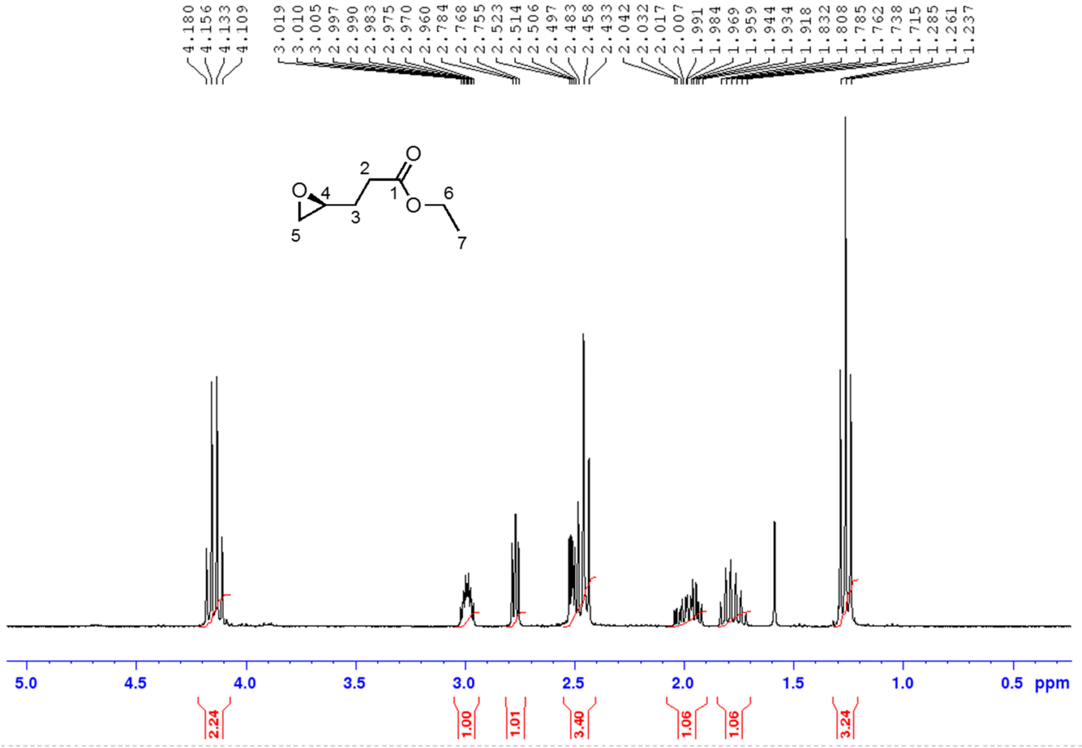

**Figure S9.** $^{1}$H-NMR spectra for (*S*)-Ethyl 4,5-epoxypentanoate (**(*S*)-1b**) crude. $^{1}$H 300 MHz in $CDCl_3$.

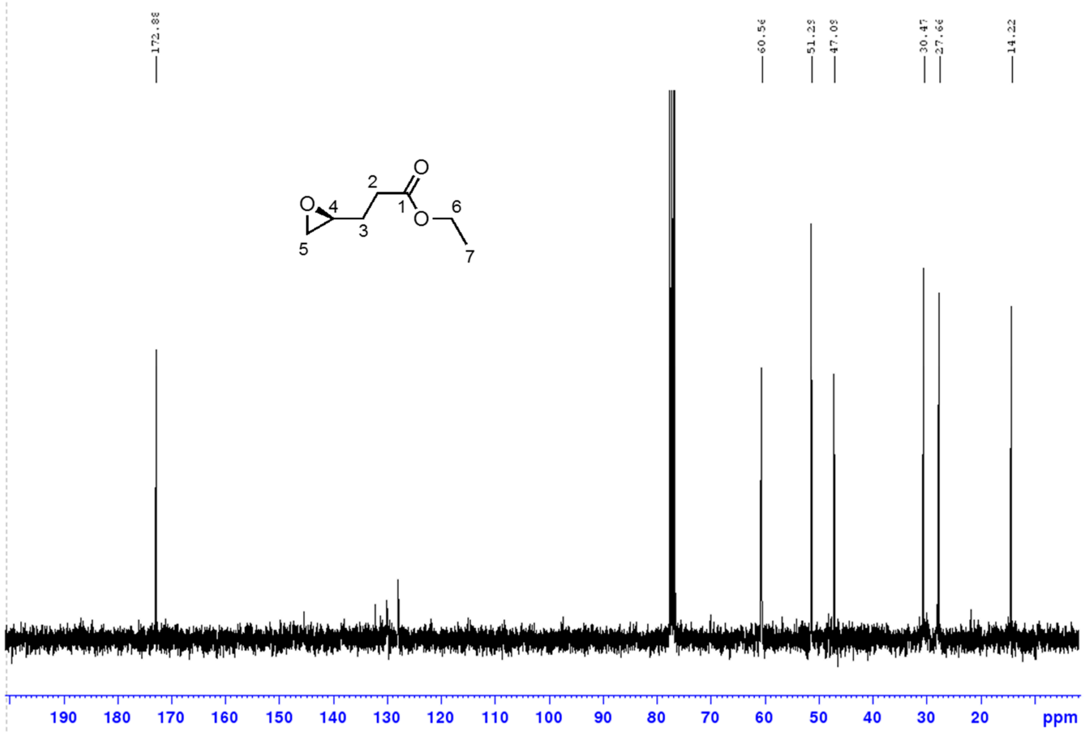


**Figure S10.** $^{13}$C-NMR spectra for (*S*)-Ethyl 4,5-epoxypentanoate (**(*S*)-1b**) crude.$^{13}$C 75 MHz in $CDCl_3$.

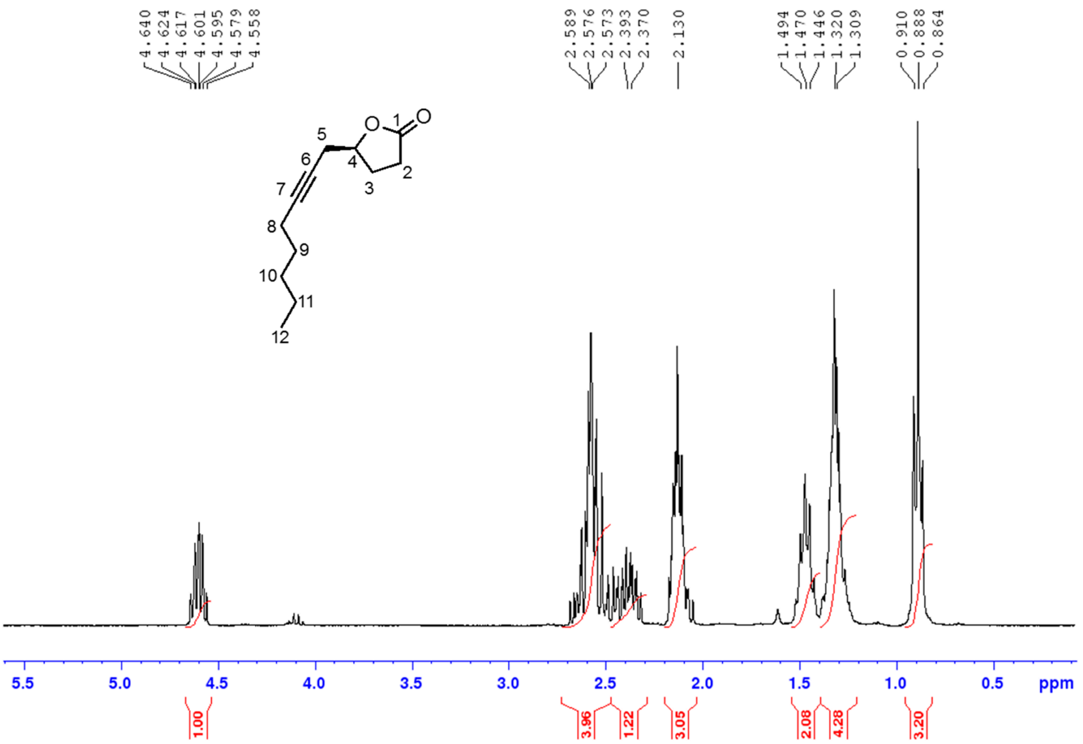


**Figure S11.** $^{1}$H-NMR spectra for (*S*)-5-(Oct-2-yn-1-yl)-γ-Butyrolactone (**5**). $^{1}$H 300 MHz in $CDCl_3$.

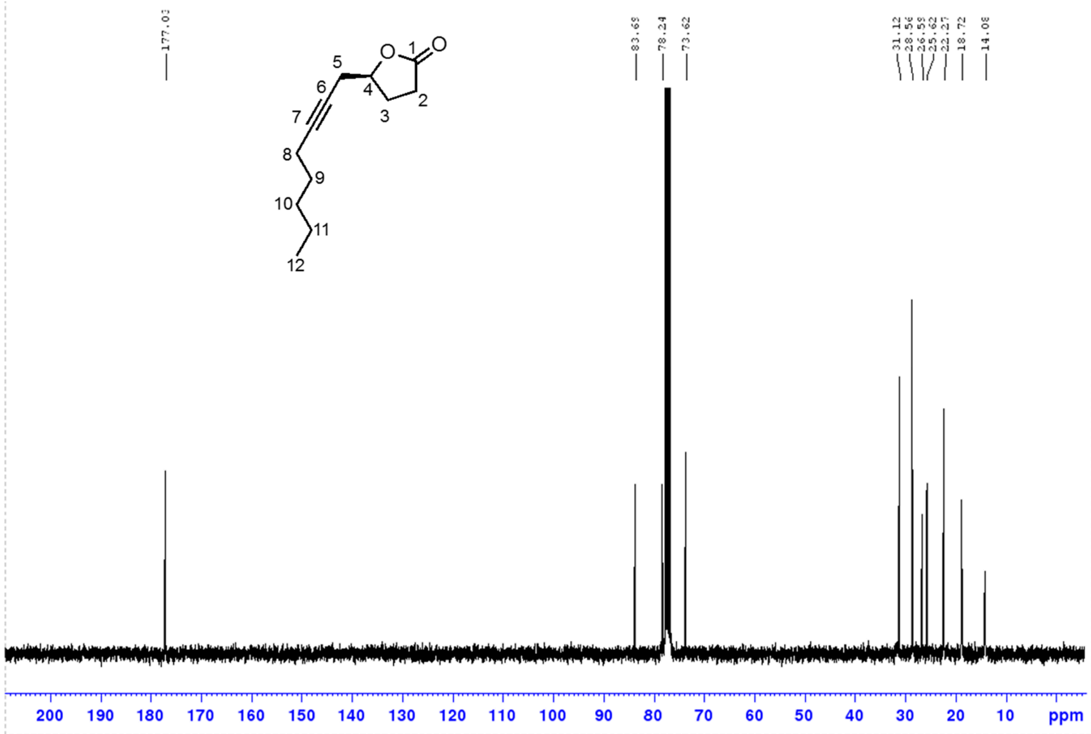


**Figure S12.** $^{13}$C-NMR spectra for (*S*)-5-(Oct-2-yn-1-yl)-γ-Butyrolactone (**5**). $^{13}$C 75 MHz in $CDCl_3$.

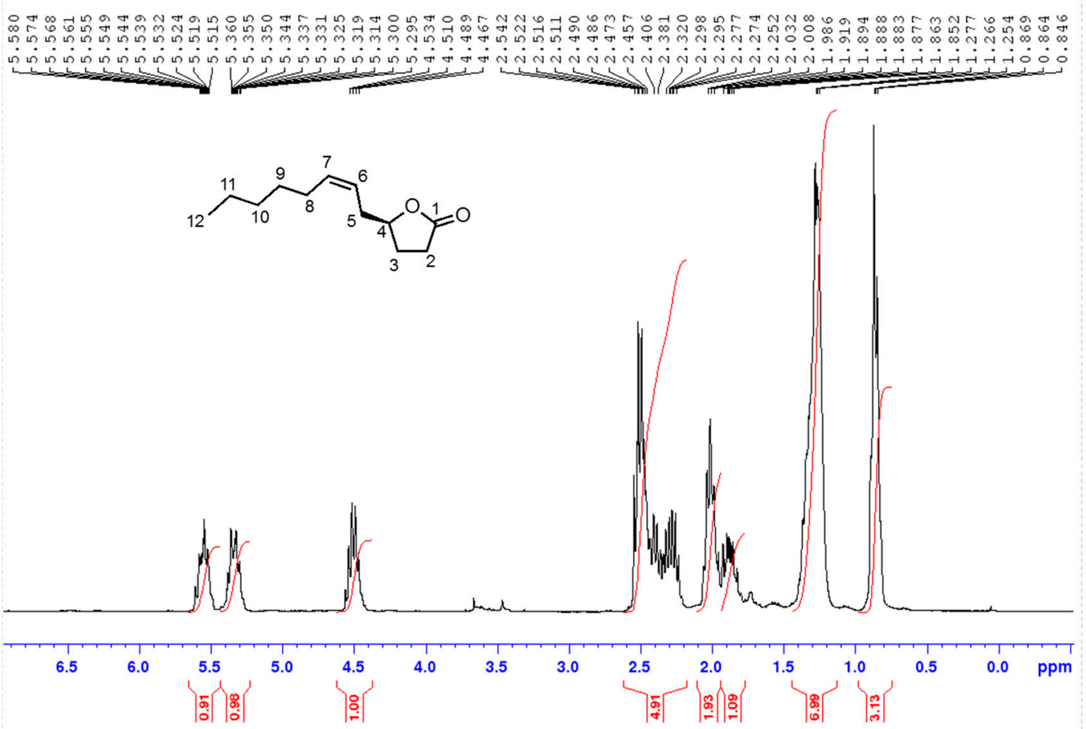


**Figure S13.** $^{1}$H-NMR spectra for (*S*)-Dairy lactone (**6**). $^{1}$H 300 MHz in $CDCl_3$.

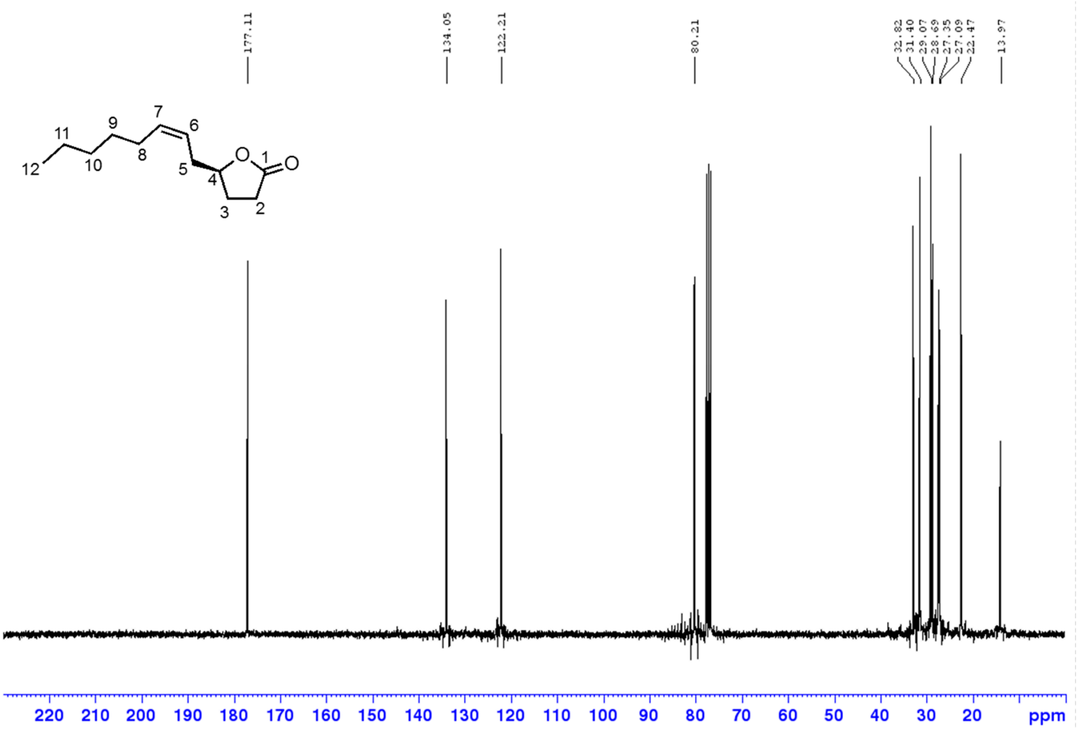


**Figure S14.** $^{13}$C-NMR spectra for (*S*)-Dairy lactone (**6**).$^{13}$C 75 MHz in $CDCl_3$.
